# Multicenter Evaluation of Different Anti-Xa Assays and Diluted Russell’s Viper Venom Time in Ex Vivo Plasma Samples from Patients Treated with Rivaroxaban or Apixaban

**DOI:** 10.3390/jcm14238274

**Published:** 2025-11-21

**Authors:** Mojca Božič Mijovski, Alenka Mavri, Jovan P. Antovic, Rickard E. Malmström, Désirée Coen Herak

**Affiliations:** 1 Department of Vascular Diseases, University Medical Centre Ljubljana, 1000 Ljubljana, Slovenia; alenka.mavri@kclj.si; 2 Faculty of Pharmacy, University of Ljubljana, 1000 Ljubljana, Slovenia; 3 Faculty of Medicine, University of Ljubljana, 1000 Ljubljana, Slovenia; 4 Department of Clinical Chemistry, Karolinska University Hospital, 171 76 Stockholm, Sweden; jovan.antovic@ki.se; 5 Department of Medicine Solna, Karolinska Institutet and Clinical Pharmacology, Karolinska University Hospital, 171 76 Stockholm, Sweden; rickard.malmstrom@ki.se; 6 Clinical Pharmacology, Karolinska University Hospital, 171 76 Stockholm, Sweden; 7 Department of Laboratory Diagnostics, University Hospital Centre Zagreb, 10000 Zagreb, Croatia; desiree.coen.herak@kbc-zagreb.hr; 8 Faculty of Pharmacy and Biochemistry, University of Zagreb, 10000 Zagreb, Croatia

**Keywords:** atrial fibrillation, rivaroxaban, apixaban, anti-Xa, laboratory methods

## Abstract

**Background/Objectives**: Different anti-Xa assays are routinely used to evaluate the plasma concentrations of direct factor X inhibitors (DXIs) rivaroxaban and apixaban, despite a lack of data on assay equivalence. Information on assay performance is particularly important at clinical decision cut-offs, such as 50 ng/mL or 30 ng/mL. The aim of this study was to evaluate the equivalence of different anti-Xa assays with the reference LC-MS/MS method for measuring rivaroxaban and apixaban concentrations in a multicenter study. In addition, the usefulness of the dRVVT as a simple coagulation test for emergency situations was evaluated. **Methods**: We included 122 patients with atrial fibrillation. Trough and peak blood samples were collected from 60 patients treated with rivaroxaban and 62 patients treated with apixaban. Rivaroxaban and apixaban plasma levels were measured by LC–MS/MS. Different anti-Xa assays were used in three laboratories to evaluate equivalence. **Results**: The concentrations in the analyzed samples ranged from 2 to 781 ng/mL for rivaroxaban and 9 to 568 ng/mL for apixaban. Only one of the anti-Xa assays gave equivalent results to LC-MS/MS for rivaroxaban, and none for apixaban. All anti-Xa assays significantly underestimated apixaban concentration, with a proportional bias between 10% and 20%. A high correlation was found between DXI concentration and dRVVT clotting time, but dRVVT was not consistently prolonged at clinically relevant DXI concentrations in plasma. **Conclusions**: Only one of the anti-Xa assays showed equivalence with LC-MS/MS for rivaroxaban, and none for apixaban. Several anti-Xa assays provided reliable results for rivaroxaban in the range of clinically relevant cut-off values, but none for apixaban, which could expose patients to a higher risk of bleeding and urgently needs further clinical research. The dRVVT test was not sensitive enough for reliable detection of clinically relevant DXI plasma concentrations and therefore cannot replace the anti-Xa assay in emergency situations.

## 1. Introduction

Rivaroxaban and apixaban are well-established direct oral factor Xa inhibitors (DXIs) widely used for the prevention and treatment of thrombotic disorders [1]. Although DXI therapy does not require routine laboratory monitoring, measuring DXI plasma concentrations can be clinically relevant in specific situations. The reference method for determining DXI levels is liquid chromatography coupled with tandem mass spectrometry (LC-MS/MS) [2]; however, this technique is not widely accessible.

Several studies have evaluated alternative approaches, using either plasma samples spiked with DXIs [3,4], samples from healthy volunteers after DXI administration [5,6], or patient samples. These investigations demonstrated that chromogenic anti-Xa assays calibrated with known DXI concentrations can serve as a suitable alternative [7,8]. High correlation coefficients between DXI concentrations and anti-Xa activity were consistently reported, supporting the widespread adoption of anti-Xa assays in routine clinical practice. Nevertheless, a strong correlation does not imply equivalence between methods. Laboratories must verify assay equivalence before clinical implementation. Commonly, this is assessed by analyzing paired results using Bland–Altman plots and estimating bias through regression techniques such as Passing–Bablok or Deming analysis [9].

Because anti-Xa assays are not standardized and significant inter-assay variability has been observed [10], equivalence with LC-MS/MS cannot be assumed. Furthermore, assay performance near clinical decision thresholds is particularly critical. For example, in patients with severe bleeding, antidote administration is recommended when drug concentrations exceed 50 ng/mL, whereas for urgent interventions with high bleeding risk, the threshold is 30 ng/mL [11,12].

Although anti-Xa assays offer shorter turnaround times compared to LC-MS/MS, they may still be impractical in emergencies due to the need for specific DXI calibrators and controls, which often restricts testing to specialized coagulation laboratories operating on limited schedules. The diluted Russell’s viper venom time (dRVVT), a simple coagulation test primarily used for lupus anticoagulant detection, has been proposed as a rapid alternative. Specifically, the phospholipid-rich dRVVT Confirm reagent, less affected by unexpected lupus anticoagulants, may provide a quick estimate of DXI anticoagulant intensity [13].

Based on these considerations, the aim of this multicenter study was to evaluate the equivalence of various anti-Xa assays with the LC-MS/MS reference method for measuring rivaroxaban and apixaban concentrations, with particular emphasis on performance near clinical decision thresholds of 50 and 30 ng/mL. Additionally, we assessed the potential utility of dRVVT as a rapid screening tool in emergency settings.

## 2. Materials and Methods

We included 122 patients with atrial fibrillation in the study: 60 patients treated with rivaroxaban (30 with 20 mg daily and 30 with 15 mg daily), and 62 patients treated with apixaban (32 with 5 mg twice daily and 30 with 2.5 mg twice daily). The detailed description of the patients can be found elsewhere [14,15]. Three trough blood samples (12 ± 1.5 h after the last apixaban dose and 24 ± 1 h after the last rivaroxaban dose) and three peak blood samples (124 ± 8 min after dosing) were collected from each patient over a period of 6 to 8 weeks; therefore, we expected 360 rivaroxaban and 372 apixaban samples. All patients signed an informed consent form in which they agreed to participate in the study. The study was approved by the Medical Ethics Committee of the Slovenian Ministry of Health. Blood samples were collected from the antecubital vein in 4.5 mL vacuum tubes containing 0.109 mol/L sodium citrate (9:1 *v*/*v*) (Becton Dickinson, Eysins, Switzerland). Platelet-poor plasma was prepared by 20 min centrifugation at 2000× *g*, aliquoted into plastic vials, and stored at ≤−70 °C until analysis. Frozen plasma aliquots were distributed from the University Medical Centre Ljubljana (Lab. A) to the Karolinska Institute (Lab. B) and the University Hospital Centre Zagreb (Lab. C) for laboratory analysis. For various reasons (missed appointments, hemolyzed samples, used samples), 358 plasma samples (out of the expected 360) with rivaroxaban and 366 plasma samples (out of the expected 372) with apixaban were available for this study. However, due to logistical problems, the anti-Xa assay with Technochrom reagent was performed in only 125 rivaroxaban samples and 116 apixaban samples.

The concentrations of rivaroxaban and apixaban were measured in plasma samples by LC–MS/MS with the lower limit of detection of 2 ng/mL [7,16].

The combination of reagents, calibrators, controls and analyzers used to evaluate anti-Xa performance in the participating centers is listed in Table 1. All anti-Xa assays were performed according to the manufacturer’s instructions, with one exception: for Berichrom Heparin, the addition of exogenous antithrombin was omitted [17]. Measuring ranges for all anti-Xa assays are provided in Supplementary Material S1. All the reported measuring ranges covered clinically relevant cut-off levels. If the DXI concentration exceeded the measuring range, a higher sample dilution was used. In addition to the anti-Xa test, dRVVT was measured in all three participating centers using the LA 2 Confirmation Reagent (Siemens Healthineers, Marburg, Germany), on a CS-2500 in Lab. A, CS-2100i in Lab. B and CS-5100 in Lab. C. APTT was measured solely in Lab. A with Pathromtin SL (Siemens Healthineers, Marburg, Germany) on a CS-2100i coagulation analyzer (Sysmex, Japan).

Statistical analysis was performed using Analyse-it for Microsoft Excel 6.10.1 (Analyse-it^®^ for Microsoft^®^ Excel 6.10.1, Leeds, UK). The laboratory results obtained were described using the median (min–max). Bland–Altman plots were created to compare pairs of results. Proportional and constant biases between LC-MS/MS and anti-Xa assays and between dRVVT results from the three participating laboratories were evaluated using Passing–Bablok regression analysis, which included the calculation of the 95% confidence interval (95% CI) for slope and intercept and expected DXI levels at clinical decision limits. Equivalence between two methods was defined as the absence of proportional bias (the 95% CI for the regression slope included 1) and the absence of constant bias (the 95% CI for the intercept included 0). The assay was considered reliable at the clinically relevant cut-off points if the 95% CI included the 30 ng/mL or 50 ng/mL value, respectively. The Spearman correlation coefficient was calculated between the DXI concentration measured by LC-MS/MS and dRVVT or APTT.

Generative artificial intelligence (GenAI) has not been used in any part of this paper.

## 3. Results

**Figure 1:**
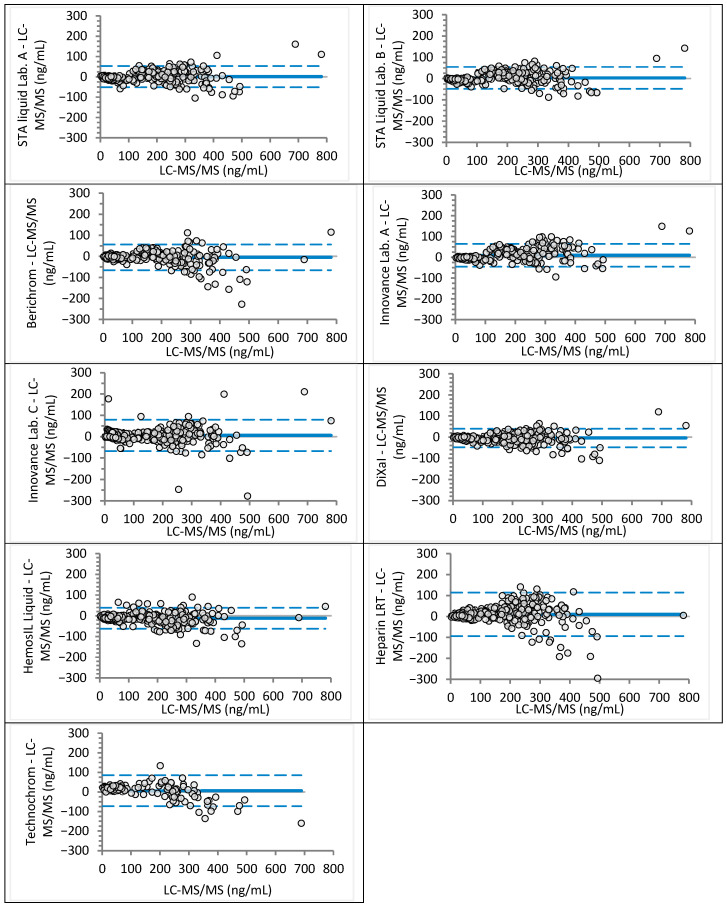
Bland–Altman analysis for rivaroxaban concentrations measured by using different anti-Xa assays in comparison to the LC-MS/MS method (absolute differences). Continuous lines depict mean difference, while dotted lines show upper and lower limits of agreement.

**Figure 2:**
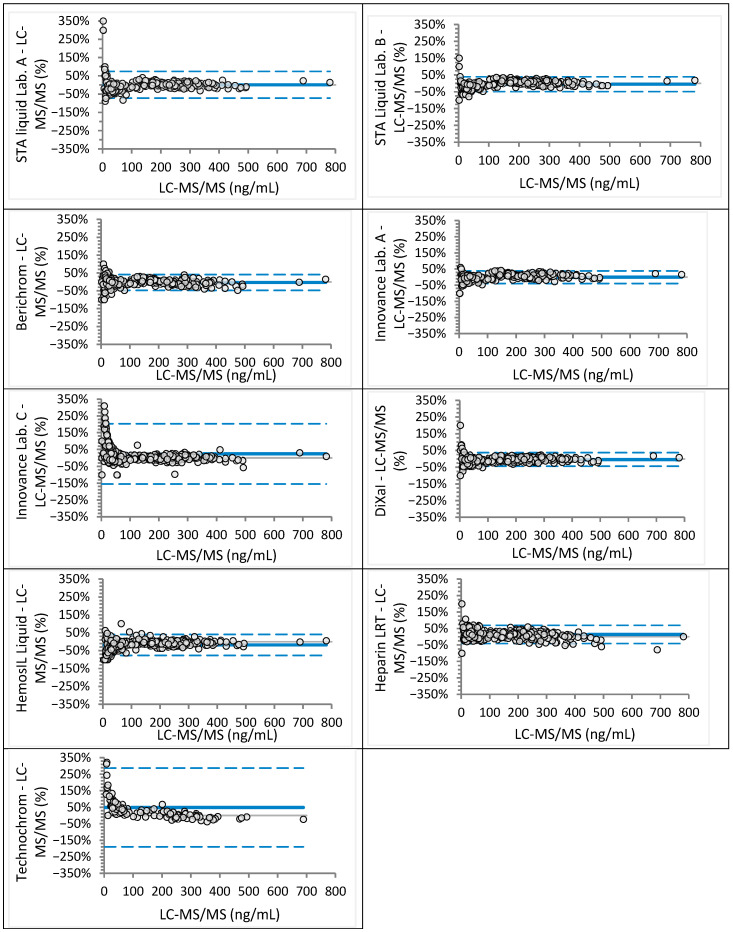
Bland–Altman analysis for rivaroxaban concentrations measured different anti-Xa assays in comparison to the LC-MS/MS method (relative differences). Continuous line depicts mean difference, while dotted lines show upper and lower limits of agreement.

**Figure 3:**
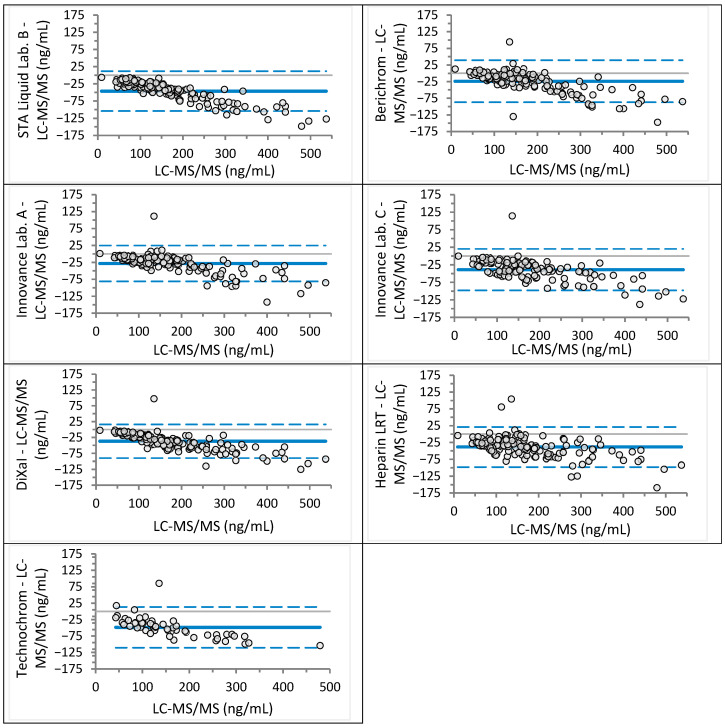
Bland–Altman analysis for apixaban concentration measured different anti-Xa assays in comparison to the LC-MS/MS (absolute differences). Continuous line depicts mean difference, while dotted lines show upper and lower limits of agreement.

**Figure 4:**
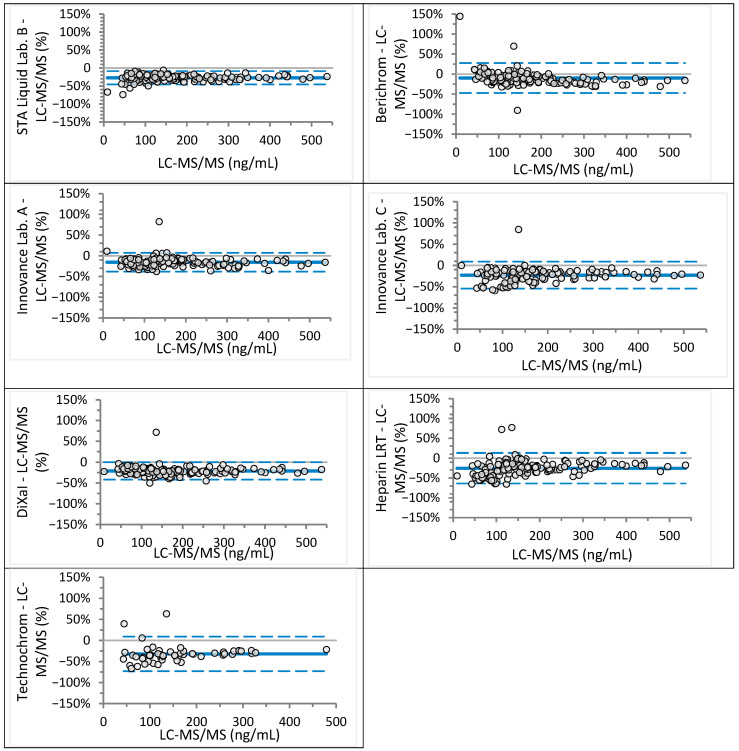
Bland–Altman analysis for apixaban concentration measured different anti-Xa assays in comparison to the LC-MS/MS (relative differences). Continuous lines depict mean difference, while dotted lines show upper and lower limits of agreement.

Analyzed sample concentrations ranged from 2 to 781 ng/mL for rivaroxaban (median 117 ng/mL) and from 9 to 568 ng/mL for apixaban (median 152 ng/mL), covering expected trough and peak levels as well as values near clinical decision cut-offs. Agreement between anti-Xa assays and LC-MS/MS was assessed visually using Bland–Altman plots (Figure 1 and Figure 2 for rivaroxaban; Figure 3 and Figure 4 for apixaban) and statistically using Passing–Bablok regression analysis (Table 2 and Table 3).

For rivaroxaban, mean differences across anti-Xa assays were generally close to zero, ranging from −12 ng/mL (HemosIL Liquid) to +10 ng/mL (Heparin LRT). The widest limits of agreement (LoA) were observed for Heparin LRT (−94 to +114 ng/mL) (Figure 1). For apixaban, all mean differences were negative, ranging from −49 ng/mL (Technochrom) to −24 ng/mL (Berichrom), with the widest LoA for Berichrom (−87 to +39 ng/mL) and Technochrom (−110 to 13 ng/mL) (Figure 3).

Passing–Bablok analysis showed that only Berichrom achieved equivalence with LC-MS/MS for rivaroxaban, with a regression slope of 0.99 (95% CI: 0.96–1.01) and an intercept of −0.84 (95% CI: −2.25 to 0.63). Innovance (Lab. A) and Heparin LRT overestimated rivaroxaban concentrations, exhibiting a proportional bias of approximately 10%. All other assays, except Berichrom and Heparin LRT, demonstrated constant bias ranging from −7.4 to +24.2 ng/mL (Table 2). Bland–Altman plots revealed marked proportional bias at low rivaroxaban concentrations (<50 ng/mL) for Innovance (Lab. C) and Technochrom (Figure 2). Regression analysis for STA-Liquid between Lab. A and Lab. B [y = 1.04 (1.03–1.05)x − 3.57 (−4.76 to −2.05)] indicated a small but statistically significant constant bias, while Innovance [y = 0.91 (0.90–0.93)x + 11.29 (6.30–15.53)] showed both proportional and constant bias. STA-Liquid (Lab. A) and Berichrom performed adequately at both clinical decision thresholds, whereas STA-Liquid (Lab. B), DiXaI, and HemosIL underestimated rivaroxaban at these cut-offs. The lowest values were obtained with HemosIL (95% CI: 20–24 ng/mL at 30 ng/mL and 40–43 ng/mL at 50 ng/mL). Innovance (Lab. C), Heparin LRT, and Technochrom tended to overestimate rivaroxaban concentrations.

For apixaban, all anti-Xa assays significantly underestimated concentrations, with proportional bias ranging from 10% to 20%. Berichrom, Innovance (Lab. C), Heparin LRT, and Technochrom also exhibited constant bias between −17.8 and +13.9 ng/mL. The regression equation for Innovance across Lab. A and Lab. C [y = 1.00 (0.98–1.02)x − 6.00 (−8.27 to −2.54)] indicated a low constant bias.

The dRVVT test showed a strong correlation with rivaroxaban concentrations across all three participating laboratories. APTT also correlated significantly with rivaroxaban, although the strength of correlation was lower compared to dRVVT. For apixaban, correlation coefficients were consistently lower than those observed for rivaroxaban in all laboratories. The weakest correlation was found between apixaban concentration and APTT (Table 4).

**Figure 5:**
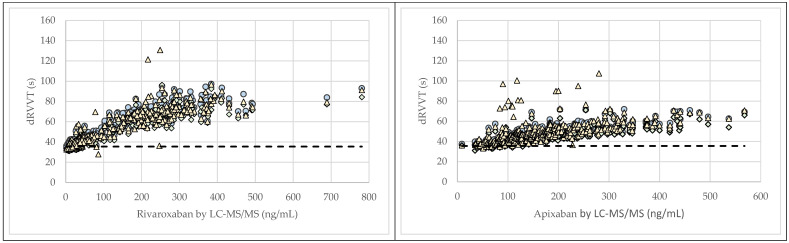
Correlation between rivaroxaban and apixaban plasma concentrations and diluted Russell’s viper venom time (dRVVT) across participating laboratories. dRVVT results are shown for Lab. A (blue circles), Lab. B (green diamonds), and Lab. C (yellow triangles). The dotted line indicates the upper reference limit for dRVVT (35.5 s).

Consistent prolongation of dRVVT beyond the upper reference limit (>35.5 s) was observed for rivaroxaban concentrations exceeding 28 ng/mL (Lab. A), 81 ng/mL (Lab. B), and 86 ng/mL (Lab. C), and for apixaban concentrations above 70 ng/mL (Lab. A), 129 ng/mL (Lab. B), and 84 ng/mL (Lab. C) (Figure 5).

Despite the use of the same reagent and analyzer family, dRVVT results were not equivalent across laboratories for either rivaroxaban or apixaban, as demonstrated by Passing–Bablok regression (Table 5) and Bland–Altman plots (Supplementary Material Figures S1 and S2).

## 4. Discussion

Our multicenter study demonstrated substantial variability in the performance of different anti-Xa assays for measuring DXI concentrations. Overall, rivaroxaban concentrations were estimated more accurately than apixaban concentrations, yet only one assay achieved equivalence with the LC-MS/MS reference method. Several assays provided reliable results for rivaroxaban near clinically relevant cut-off values, but none did so for apixaban. Although dRVVT showed a strong correlation with DXI concentrations, it failed to detect rivaroxaban or apixaban at clinically relevant thresholds and therefore cannot replace anti-Xa testing in emergency settings.

To our knowledge, this is the first study to systematically evaluate agreement and equivalence between multiple anti-Xa assays and the LC-MS/MS reference method for rivaroxaban and apixaban measurement. Differences between rivaroxaban concentrations obtained by anti-Xa and LC-MS/MS were generally centered around zero, but the widest limits of agreement (LoA) were observed for Heparin LRT, indicating lower accuracy and reliability—confirmed by regression analysis. Previous studies reported narrower LoAs for Heparin LRT [18], likely due to fewer samples with concentrations above 300 ng/mL, which contributed significantly to variability in our dataset. For all other assays, LoAs were narrower than those reported in a similar study [18], likely reflecting our larger sample size and the use of LC-MS/MS rather than HPLC-UV as the comparative method.

Berichrom was the only assay demonstrating equivalence with LC-MS/MS according to Passing–Bablok analysis, whereas other assays exhibited proportional or constant bias, or both. This contrasts with Cini et al. [18], who reported overestimation of rivaroxaban by Berichrom, attributed to exogenous AT addition. In our modified protocol, AT was replaced by buffer, and previous work confirmed no significant difference between protocols [17]. Performance differences are more plausibly explained by calibrator choice (Hyphen Biomed vs. Stago) and analyzer type (BCS vs. CS-2500). Calibrator influence was evident in Innovance assays performed in two laboratories: identical analyzers and protocols yielded different bias patterns depending on calibrator source (Stago vs. Technoclone) (Table 2).

Higher bias in anti-Xa results for rivaroxaban concentrations below 50 ng/mL has been reported previously [7,10,19]. In our study, two assays calibrated with Technoclone rivaroxaban calibrators (Innovance Lab. C and Technochrom) showed marked disagreement at concentrations below 50 ng/mL and overestimated rivaroxaban at clinically relevant cut-offs (Figure 2, Table 2). Whether this discrepancy reflects calibrator composition or assay protocol (e.g., suboptimal dilution in the low range) warrants further investigation. Similarly, DiXaI, which also used separate calibration curves for high and low ranges, significantly underestimated rivaroxaban at clinically relevant cut-offs, indicating that dual-range calibration is not inherently advantageous. Berichrom performed best across both ranges, but its discontinuation necessitates alternatives. Based on our findings, Innovance Heparin combined with STA-Rivaroxaban Calibrator and CS-2500 analyzer appears to be the most suitable option.

Unexpectedly, all anti-Xa assays underestimated apixaban concentrations, as shown by both Bland–Altman and Passing–Bablok analyses. Cini et al. [18] reported similar findings for STA-Liquid, Heparin LRT, and DiXaI, but not for Berichrom, Innovance, or Technochrom. These discrepancies may reflect differences in reference methods (HPLC-UV vs. LC-MS/MS) and assay-calibrator-analyzer combinations. Underestimation of apixaban poses a clinical risk, as patients may undergo invasive procedures or be denied antidote administration based on falsely low results. In some cases, measured apixaban concentrations were only half of the actual value (Table 3).

Analytical variability can arise from differences in calibrator composition, reagent batches, and analyzer types. The absence of an international reference plasma standard for rivaroxaban and apixaban precludes traceability for all calibrators used. All commercial calibrators available are prepared by spiking pooled plasma with DXIs, minimizing matrix effects and non-commutability. Samples were processed under standardized conditions across laboratories, reducing operator-related variability. All analyzers relied on photometric detection, but differences in signal processing and reaction conditions likely contributed to discrepancies. External quality schemes (e.g., INSTAND, ECAT) confirm that reported DXI concentrations vary by reagent-analyzer combination. These sources of variation remain insufficiently studied. While laboratories could theoretically derive corrected cut-offs from our data, we strongly advise against this approach and urge manufacturers to improve standardization or harmonization of anti-Xa assays.

Although dRVVT correlated strongly with DXI concentrations—more so than APTT—it was not consistently prolonged at clinically relevant levels. Moreover, inter-laboratory variability persisted despite identical reagents and analyzer families, likely due to the absence of calibration. Thus, dRVVT cannot be recommended as a reliable alternative for rapid DXI assessment in emergency settings.

One limitation of our study is that single rather than duplicate measurements were performed for all samples. However, given the large number of measurements, the likelihood of missing a systematic bias is minimal. Another limitation was the significantly smaller number of samples analyzed with the Technochrom reagent, which reduces the statistical power of findings related to this assay.

The strengths of our study include direct comparison with the LC-MS/MS reference method for rivaroxaban and apixaban measurement, the large number of patient samples from routine clinical practice covering a wide concentration range, and the inclusion of multiple anti-Xa assays with different calibrator combinations, reflecting real-world conditions. Testing patient plasma samples captures the biological and pathological variability encountered in clinical practice, providing a realistic assessment of assay robustness. Furthermore, results derived from patient samples are directly applicable to clinical decision-making. Unlike reference plasma, patient samples may contain interfering substances (e.g., drugs, antibodies, proteins), which helps identify potential limitations of the assays.

In conclusion, regardless of reagent, calibrator, control, or analyzer combination, all anti-Xa assays estimated rivaroxaban concentrations more accurately than apixaban concentrations. However, only one assay demonstrated equivalence with the LC-MS/MS reference method for rivaroxaban. Several assays provided reliable results for rivaroxaban near clinically relevant cut-off values, but none did so for apixaban, which may expose patients to an increased risk of bleeding—a concern that warrants urgent further clinical investigation. The dRVVT test, despite its simplicity, was insufficiently sensitive to detect clinically relevant rivaroxaban and apixaban concentrations and therefore cannot replace anti-Xa testing in emergency situations.

## Figures and Tables

**Table 1:** Analyzer, anti-Xa assay, calibrator and control combinations used at participating laboratories.

Laboratory	Analyzer (Manufacturer)	Anti-Xa Assay (Manufacturer)	Rivaroxaban Calibrator and Control Set (Manufacturer)	Apixaban Calibrator and Control Set (Manufacturer)
Lab. A	CS-2500 (Sysmex, Kobe, Japan)	STA-Liquid Anti-Xa (Diagnostica Stago, Asnières sur Seine, France)	STA-Rivaroxaban Calibrator, STA-Rivaroxaban Control (Diagnostica Stago)	/
		Berichrom Heparin (Siemens Healthineers, Marburg, Germany)		STA-Apixaban Calibrator, STA-Apixaban Control (Diagnostica Stago)
		Innovance Heparin (Siemens Healthineers)		
		Biophen DiXaI (Hyphen Biomed, Neuville-sur-Oise, Francija)	Biophen Rivaroxaban Plasma Calibrator, Biophen Rivaroxaban Calibrator Low, Biophen Rivaroxaban Control Plasma, Biophen Rivaroxaban Control Low (Hyphen Biomed)	Biophen Apixaban Plasma Calibrator, Biophen Apixaban Calibrator Low, Biophen Apixaban Control Plasma, Biophen Apixaban Control Low (Hyphen Biomed)
		HemosIL Liquid Anti-Xa (Werfen, Bedford, MA, United States)	HemosIL Rivaroxaban Calibrators, HemosIL Rivaroxaban Controls (Werfen)	/
Lab. B	CS-2100i (Sysmex)	STA-Liquid Anti-Xa (Diagnostica Stago)	STA-Rivaroxaban Calibrator, STA-Rivaroxaban Control (Diagnostica Stago)	STA-Apixaban Calibrator, STA-Apixaban Control (Diagnostica Stago)
Lab. C	CS-5100 (Sysmex)	Innovance Heparin (Siemens Healthineers)	Technoview Rivaroxaban CAL High Set, Technoview Rivaroxaban CAL Set, Technoview Rivaroxaban High, Medium and Low Control (Technoclone)	Technoview Apixaban CAL Set, Technoview Apixaban Low and High Control (Technoclone, Vienna, Austria)
		Biophen Heparin LRT (Hyphen Biomed)		
	Ceveron s100 (Technoclone)	Technochrom anti-Xa (Technoclone)		

**Table 2:** Passing–Bablok regression analysis for rivaroxaban with 95% confidence intervals (95% CI) at clinical cut-off of 30 and 50 ng/mL.

Anti-Xa Assay (ng/mL)	Regression Equation	Slope 95% CI	Intercept 95% CI	95% CI at 30 ng/mL	95% CI at 50 ng/mL
STA Liquid Lab. A	y = 1.02x − 2.77	1.00 to 1.05	−4.45 to −1.00	27–30	47–50
STA Liquid Lab. B	y = 1.06x − 5.68	1.03 to 1.08	−6.91 to −4.00	25–27	46–48
Berichrom	y = 0.99x − 0.84	0.96 to 1.01	−2.25 to 0.63	28–30	47–50
Innovance Lab. A	y = 1.09x − 4.31	1.07 to 1.12	−5.38 to −3.18	28–29	49–51
Innovance Lab. C	y = 1.00x + 7.00	0.97 to 1.03	2.32 to 10.44	33–40	53–59
DiXaI	y = 1.00x − 1.95	0.97 to 1.01	−3.22 to −1.07	27–29	47–48
HemosIL Liquid	y = 0.98x − 7.38	0.95 to 1.00	−9.18 to −5.53	20–24	40–43
Heparin LRT	y = 1.11x + 1.30	1.07 to 1.14	−0.05 to 3.39	34–36	56–59
Technochrom	y = 0.91x + 24.18	0.85 to 0.96	19.68 to 27.74	48–55	66–73

**Table 3:** Passing–Bablok regression analysis for apixaban, with 95% confidence intervals (95% CI) at clinical cut-off of 30 and 50 ng/mL.

Anti-Xa Assay (ng/mL)	Regression Equation	Slope 95% CI	Intercept 95% CI	95% CI at 30 ng/mL	95% CI at 50 ng/mL
STA Liquid Lab. B	y = 0.79x − 0.58	0.77 to 0.81	−3.02 to 1.81	21–25	37–41
Berichrom	y = 0.18x + 13.86	0.79 to 0.84	11.55 to 17.06	36–41	53–57
Innovance Lab. A	y = 0.89x − 1.90	0.87 to 0.91	−4.40 to 0.43	23–27	40–44
Innovance Lab. C	y = 0.87x − 6.26	0.85 to 0.90	−10.19 to −3.01	17–23	34–40
DiXaI	y = 0.81x − 0.05	0.79 to 0.83	−2.35 to 2.20	22–26	39–42
Heparin LRT	y = 0.91x − 15.97	0.88 to 0.94	−21.38 to −11.92	7–15	25–32
Technochrom	y = 0.81x − 17.79	0.76 to 0.85	−26.69 to −11.43	0–11	16–27

**Table 4:** Correlation between rivaroxaban or apixaban concentration and dRVVT or APTT. Spearman’s rhos with 95% confidence intervals (95% CI) are given.

Assay	Rivaroxaban Concentration	Apixaban Concentration
dRVVT Lab. A	0.928 (0.912–0.942)	0.844 (0.811–0.872)
dRVVT Lab. B	0.924 (0.906–0.938)	0.834 (0.798–0.863)
dRVVT Lab. C	0.898 (0.875–0.917)	0.742 (0.690–0.786)
APTT	0.783 (0.737–0.821)	0.327 (0.229–0.418)

**Table 5:** Passing–Bablok regression for dRVVT performed at three centers with 95% confidence intervals (95% CI).

dRVVT (s)—Rivaroxaban	Regression Equation	Slope 95% CI	Intercept 95% CI
Lab. A and Lab. B	y = 0.91x +0.12	0.90 to 0.93	−0.43 to 0.63
Lab. A and Lab. C	y = 0.97x − 0.93	0.96 to 0.98	1.56 to −0.44
Lab. C and Lab. B	y = 0.94x + 1.06	0.92 to 0.95	0.39 to 1.68
**dRVVT (s)—Apixaban**			
Lab. A and Lab. B	y = 0.87x + 2.91	0.85 to 0.89	2.05 to 3.85
Lab. A and Lab. C	y = 1.06x − 3.20	1.03 to 1.10	4.97 to −1.65
Lab. C and Lab. B	y = 0.82x + 5.66	0.79 to 0.84	4.50 to 6.97

## Data Availability

The raw data supporting the conclusions of this article will be made available by the authors on request.

## Supplementary Materials

The following supporting information can be downloaded at: https://www.mdpi.com/article/10.3390/jcm14238274/s1.

## Abbreviations

The following abbreviations are used in this manuscript:

APTT	activated partial thromboplastin time
DXI	direct factor X inhibitors
dRVVT	diluted Russell’s viper venom time
LC-MS/MS	liquid chromatography with tandem mass spectrometry
