# Supplementary material for: Multicenter Evaluation of Different Anti-Xa Assays and Diluted Russell’s Viper Venom Time in Ex Vivo Plasma Samples from Patients Treated with Rivaroxaban or Apixaban
Source: J Clin Med. 2025 Nov 21;14(23):8274. doi: 10.3390/jcm14238274 (PMC12692558; doi:10.3390/jcm14238274)
Supplement: Supplementary file 1 [file jcm-14-08274-s001.zip › jcm-3959101-supplementary.pdf]

Supplementary Material S1

Table S1. Manufacturer's claims on measuring ranges of anti-Xa assays for rivaroxaban and apixaban.

	Measuring range for rivaroxaban (ng/mL)	Measuring range for apixaban (ng/mL)
STA-Liquid Anti-Xa (Diagnostica Stago)	25 – 500	23 – 500
Berichrom Heparin (Siemens Healthineers)	not reported	not reported
Innovance Heparin (Siemens Healthineers)	20 – 350	20 – 350
Biophen DiXaI (Hyphen Biomed)	0 – 500	0 – 600
HemosIL Liquid Anti-Xa (Werfen)	20 – 1000	15 – 1000
Biophen Heparin LRT (Hyphen Biomed)	0 – 600	0 - 600
Technochrom anti-Xa (Technoclone)	10 – 700	20 – 700

Supplementary Material S2

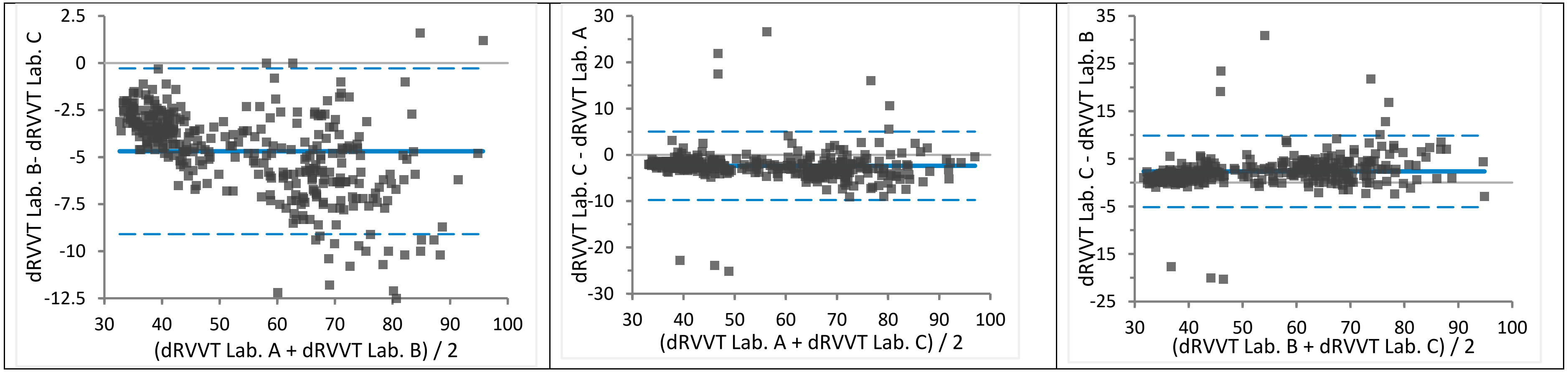

Figure S1. Bland-Altman analysis of the dRVVT between laboratories for rivaroxaban. Continuous lines depict mean difference, while dotted lines show upper and lower limits of agreement.

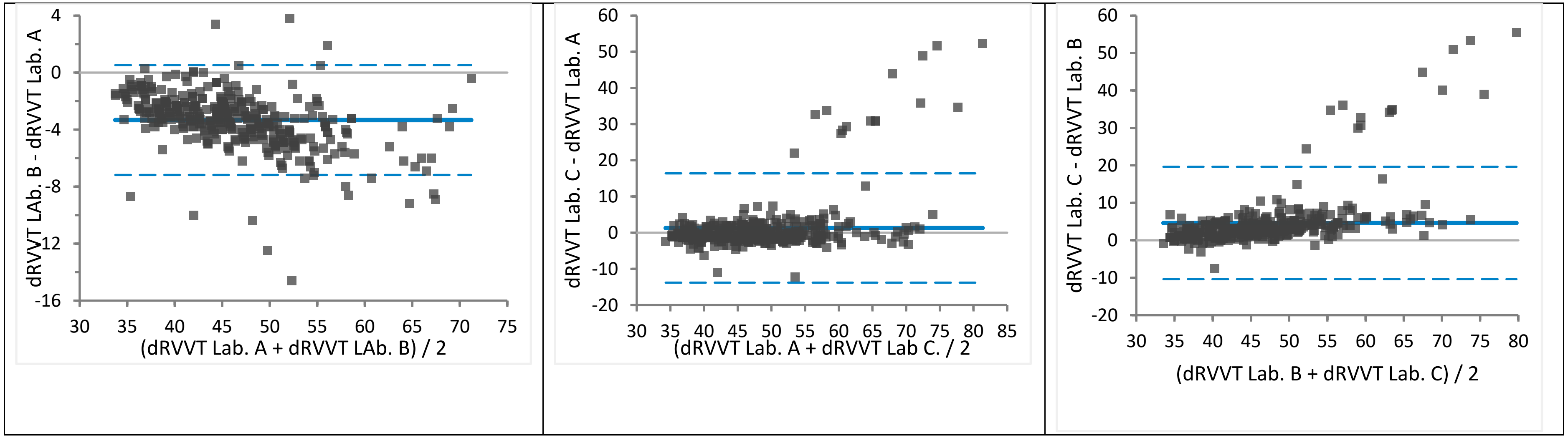

Figure S2. Bland-Altman analysis of the dRVVT between laboratories for apixaban. Continuous lines depict mean difference, while dotted lines show upper and lower limits of agreement.
